# Wireless dielectrophoresis trapping and remote impedance sensing via resonant wireless power transfer

**DOI:** 10.1038/s41467-022-35777-2

**Published:** 2023-01-06

**Authors:** Christopher T. Ertsgaard, Minki Kim, Jungwon Choi, Sang-Hyun Oh

**Affiliations:** Department of Electrical and Computer Engineering, University of Minnesota, Minneapolis, MN 55455 USA

**Keywords:** Electrical and electronic engineering, Lab-on-a-chip, Characterization and analytical techniques

## Abstract

Nearly all biosensing platforms can be described using two fundamental steps—collection and detection. Target analytes must be delivered to a sensing element, which can then relay the transduced signal. For point-of-care technologies, where operation is to be kept simple, typically the collection step is passive diffusion driven—which can be slow or limiting under low concentrations. This work demonstrates an integration of both active collection and detection by using resonant wireless power transfer coupled to a nanogap capacitor. Nanoparticles suspended in deionized water are actively trapped using wireless dielectrophoresis and positioned within the most sensitive fringe field regions for wireless impedance-based detection. Trapping of 40 nm particles and larger is demonstrated using a 3.5 V_RMS_, 1 MHz radiofrequency signal delivered over a distance greater than 8 cm from the nanogap capacitor. Wireless trapping and release of 1 µm polystyrene beads is simultaneously detected in real-time over a distance of 2.5 cm from the nanogap capacitor. Herein, geometric scaling strategies coupled with optimal circuit design is presented to motivate combined collection and detection biosensing platforms amenable to wireless and/or smartphone operation.

## Abstract (web summary)

The authors use resonant wireless power transfer to remotely trap and release nanoparticles in solution and detect their presence as a shift in impedance in real-time. This is accomplished using the extreme field confinement of a nanogap capacitor.

## Introduction

With the SARS-CoV-2 global pandemic, the existential threat of future pathogenic calamities motivates an increased development for at-home, point-of-care (POC), diagnostic technology. At the core, most POC technology involves collection of suspended particles (e.g., virus, bacteria, protein, etc.) towards a sensing element, followed by transducing a read-out signal for detection. For such devices to practically serve a broad community, not only must they be accurate and fast, but they must be accessible. With the prevailing distribution of smartphone technology, the growing majority has access to a portable power source and computational device for interfacing with POC technology. Further, as wireless power transfer (WPT) technology has continued to mature^1–4^, more sophisticated automation can be realized remotely. For these reasons, development of wireless detection strategies within the biological sector continues to gain broad interest, including demonstrations of continuous wireless monitoring of bacteria and cancer cells^5–7^, biometric monitoring via wireless wearable sweat sensors^8^, and wireless feedback from surgical implants^9^.

While wireless POC poses great advantages, the transfer of target analytes to sensing surfaces has remained rather primitive. Generally, the approach is a diffusion-based transfer process, which results in random analyte placement (filling only a fraction of the sensing elements) and can be slow if the concentration is low^10,11^. Wireless manipulation of microstructures has been demonstrated for micro-robotic operations^12,13^. However, these demonstrations required the target particles to be uniquely engineered to achieve the desired manipulation. Alternatively, dielectrophoresis (DEP) actuation can provide active and rapid particle manipulation of arbitrary suspended particles using radiofrequency (RF) signals that do not require a particular charge, magnetic moment, or chemical tag^14^. Instead, an electric-field gradient driven at an RF frequency is used to induce a local dipole moment about the particle and collect them within the fringe field regions surrounding the working electrodes. As suspended particles become trapped within these fringe field volumes, they displace the ambient solution and cause a change in reflected impedance^15^. For these reasons, groups have explored various DEP-based actuation and sensing strategies to utilize these complementary mechanisms^16,17^. However, these schemes typically require a sufficiently large voltage (10–100 V) for trapping small particles and thus make detecting a small signal change from the trapped particles difficult.

**Fig. 1:**
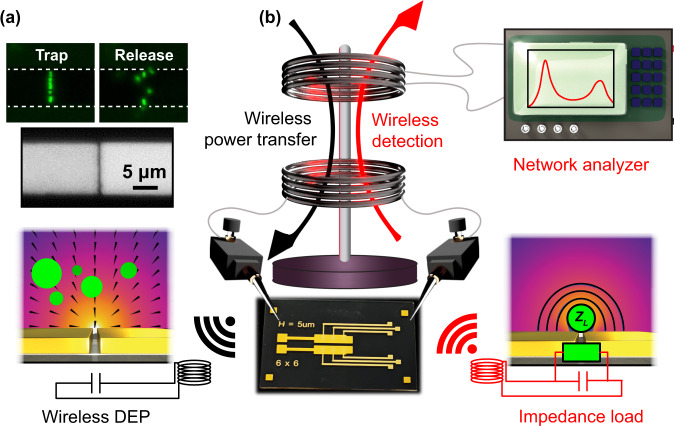
Experiment concept: real-time wireless collection and detection. **a** A microscope image of a single coplanar nanogap used for trap and release experiments. Fluorescent images demonstrate wireless manipulation of 200 nm polystyrene particles. **b** A concept diagram of our experimental setup. As a primary and secondary coil inductively couple RF power to a nanogap capacitor, strong electric-field gradients create a polarization force that collects particles towards the nanogap (lower left, “Wireless DEP”). At this junction, the confined electric fields are sensitive to changes in the dielectric load (lower right, “Impedance load”), resulting in a discernable shift in reflected impedance, which is measured wirelessly across our nanogap array device (center) using a network analyzer (upper right).

Fortunately, Moore’s law of scaling has rendered nanofabrication techniques capable to address this issue. Since DEP is a geometrically scalable technique, particles of decreasing size can be trapped by instead confining the field gradient rather than increasing the driving voltage. As a rule of thumb, the distance of separation between DEP electrodes should be of the order in size of the particles that are to be trapped. With modern fabrication techniques, gaps and field gradients on the scale of tens of nanometers are readily achieved, allowing for collection of nanoparticles of comparable size. With the efficient scaling of a nanocapacitor used for DEP, only one low-volt signal is necessary for simultaneous trapping and detection of suspended particles. This advantage is enhanced when utilizing a resonant tank circuit in which the parasitic capacitance of the device is canceled by inductive reactance resulting in larger voltage gains for a given power. This increases the efficiency of particle collection and creates a higher quality factor resonator for heightened sensitivity when detecting impedance changes made from the trapped particles. Additionally, the same resonant inductor can serve as an antenna for WPT via inductive coupling^18^. Some work regarding wireless DEP manipulation has been explored^19,20^. but instead the particles were repelled from the sensitive fringe fields using negative DEP and thus wireless detection was not implemented. Instead, we demonstrate low-volt modulation using a nanogap capacitor with WPT and a positive DEP architecture to collect the particles at the fringe fields for simultaneous wireless detection (Fig. 1). We believe active collection to be a necessary advancement to future POC technology and thus present a simultaneous wireless collection and detection method to inspire more targeted POC applications.

## Results

### Motivating the wireless DEP architecture

As mentioned above, the RF frequency is important for driving particle manipulation within a dielectric medium. If the frequency of the signal is such that this induced dipole has time to respond, the particle will migrate towards locations with increasing electric-field gradient. If the particle cannot respond as compared to the surrounding medium, the surrounding liquid solution fills these high-field regions resulting in the particle being expelled. The parameter determining the direction a particle moves along the E-field gradient is contained within the frequency dependent, complex Clausius-Mossotti factor (CMF), see Supporting Information. The sign on the real part of the CMF indicates whether the particle will move towards (positive DEP or pDEP) or away (negative DEP or nDEP) from increasing E-field gradients. The CMF of polystyrene beads in deionized (DI) water, which were used as model particles in all experiments, was plotted as a function of frequency (Fig. S1) and the crossover frequency from pDEP to nDEP is estimated to be 2.58 MHz, see supporting information for details. For this work, it is desired to precisely position particles at the nanogap capacitor where both the image plane of a microscope and the sensitive fringe field regions for RF sensing exist. Therefore, the pDEP domain will be the target for this application.

By considering a spherical particle with a radius, *a*, suspended in a medium with a dielectric constant, *ε*_*m*_, the DEP force equation (Eq. 1) strongly depends on the gradient of the E-field, *∇|E* | .

1 $${{{{{{\bf{F}}}}}}}_{{{{{{\rm{DEP}}}}}}}=\pi {\varepsilon }_{m}{a}^{3}{{{{{\rm{Re}}}}}}\left\{{f}_{{CM}}^{*}\left(\omega \right)\right\}{{{{{\boldsymbol{\nabla }}}}}}{\left|E\right|}^{2}$$

As mentioned prior, frequency dependent CMF, *f*_CM_*(*ω*), determines the direction of the DEP force. According to Eq. 1, the particle size has a higher power influence on the trapping force (i.e., cubic) as compared to the gradient (i.e., quadratic) and thus ever increasing voltages are needed for a given gradient to manipulate smaller particles. Conversely, the gradient can be increased to compensate instead by using extreme nanogap scaling of the working electrodes. This can then offer manipulation of smaller particles (e.g., tens of nanometers) at lower voltages—even as low as digital transistor-transistor logic or weakly transmitted RF signals.

**Fig. 2:**
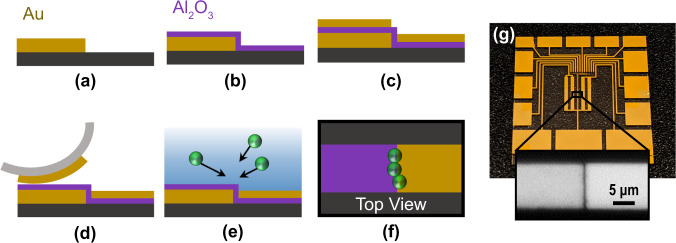
Coplanar nanogap fabrication. Cross-sectional, fabrication steps for the coplanar electrode DEP device. **a** Step 1: Au is patterned to define the first electrode. **b** Step 2: A conformal 20 nm Al_2_O_3_ layer is deposited using ALD to define the electrode gap along the edge of the first electrode. **c** Step 3: Au is evaporated to define the second electrode. **d** Step 4: Excess top Au is removed using adhesive tape. **e** Solution with the suspended analytes is applied over the whole trap. **f** Top view of the completed coplanar trap. The ALD Al_2_O_3_ layer covers the left electrode and the gap between electrodes is defined by the thickness of the ALD Al_2_O_3_ layer. **g** A final device image of a coplanar array of DEP devices. (inset) Microscope image of a single coplanar nanogap junction.

**Fig. 3:**
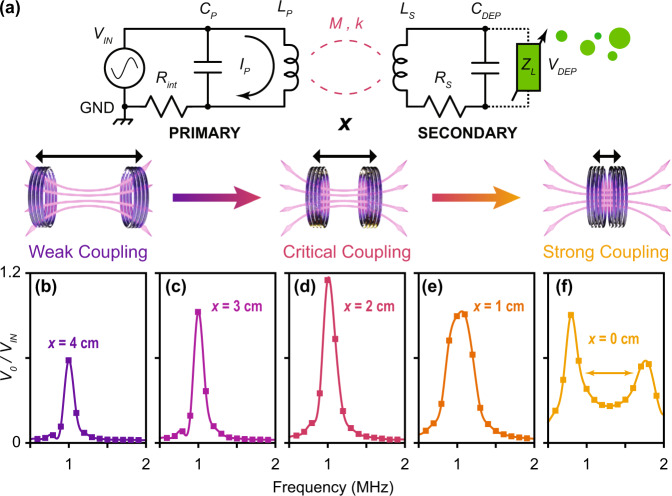
Circuit architecture and optimal coupling regime. **a** A circuit diagram describing our wireless DEP and impedance sensing architecture. Power is inserted at V_IN_ (using a function generator with an internal resistance R_int_) and drives current, I_P_, through the primary inductor L_P_, relative to ground, GND. This power is coupled to the secondary inductor, L_S_, via mutual inductance, M, with a coupling coefficient, k, which depends on the distance of separation, x, between the two solenoid inductors. A variable load impedance, Z_L_, models the trapping of particles and can be detected on the primary circuit as a change in reflected impedance. The primary capacitor, C_P_, and device capacitance, C_DEP_, define the resonant frequency needed for optimal coupling. **b–f** A demonstration of strong coupling and the importance in finding the optimal coupling regime for efficient wireless power transfer. The wireless voltage gain over the coplanar nanogap was measured as a function of frequency as the distance, x, between the primary and secondary coil was decreased. As the distance was reduced, a larger voltage gain was observed until frequency splitting occurred due to strong coupling. This splitting in resonance from the coupled primary and secondary circuit effectively reduces the voltage at the target 1 MHz frequency needed for wireless particle trapping.

To demonstrate, a 20 nm Al_2_O_3_ gap separation between two Au electrodes were fabricated using atomic layer deposition (ALD) to precisely control the electrode spacing and alignment over relatively larger areas (mm^2^), see Methods section below. Due to the potentially large parasitic capacitance from nanometer separated working electrodes, a coplanar electrode arrangement was initially used to minimize the capacitance, measured at 22.2 ± 0.8 pF in DI water. The general fabrication process is provided in the Supplementary Materials (Fig. 2). This nanogap capacitor was then integrated with an optimal WPT circuit where a primary circuit (i.e. contains the power supply) was designed to efficiency couple power to this secondary DEP circuit, see Fig. 3a. Inductive coupling between a primary inductor, *L*_*P*_, and secondary inductor *L*_*S*_, is used. The mutual inductance of the inductor pair, *M*, can be defined as the following:

2 $$M=k\sqrt{{L}_{P}{L}_{S}}$$

where, *k*, is the coupling coefficient and will vary between 0 and 1 depending on the fractional overlap of the two inductors’ magnetic flux^21,22^. This coupling coefficient will grow exponentially as the separation between the two coils, *x*, is reduced until strong-coupling effects occur^23,24^. If the secondary inductor is applied across the DEP device, the voltage, *V*_*DEP*_, dropped over the DEP device will then depend on the mutual inductance (Eq. 2) and the current carrying power through the primary inductor, *I*_*P*_, see Eq. 3 and Fig. 3a.

3 $${V}_{{{{{{{\mathrm{DEP}}}}}}}}=\frac{M}{{C}_{{{{{{{\mathrm{DEP}}}}}}}}{Z}_{s}}{I}_{P}$$

Here, the DEP device has a capacitance, *C*_*DEP*_, and forms a secondary LCR circuit with impedance, *Z*_*S*_, see Supplementary Materials Equation S6. This impedance will be minimized and thus the DEP trapping force maximized when the angular operating frequency meets the circuit’s resonant condition below:

4 $${\omega }_{0}=\frac{1}{\sqrt{{L}_{S}{C}_{{{{{{{\mathrm{DEP}}}}}}}}}}$$

However, the current through the primary inductor, *I*_*P*_, contains a component of reflected impedance, *Z*_*r*_, which is inversely proportional to *Z*_*S*_, see Eq. 5.

5 $${Z}_{r}=\frac{{\left(\omega M\right)}^{2}}{{Z}_{s}}$$

Therefore, it is advantageous to design a primary parallel LC circuit to promote current gains, *I*_*P*_, through the transmitting primary inductor to compensate, see Supplementary Materials Equation S7. While this reflected impedance can limit the coupling of power and thus the DEP trapping force (Eqs. 1 and 3), it also enables wireless detection of trapped particles.

The total input impedance, *Z*_*IN*_, of the combined wireless circuit is:

6 $${Z}_{{IN}}={R}_{{{{{{\rm{int}}}}}}}+\frac{j{X}_{{CP}}\left({{jX}}_{{LP}}+{Z}_{r}\right)}{j{X}_{{CP}}+{{jX}}_{{LP}}+{Z}_{r}}$$

which includes the power source’s internal resistance, *R*_int_, (typically standardized to 50 Ω) and the reactance of the primary inductor and capacitor, *X*_*LP*_ and *X*_*CP*_, respectively. Trapped particles cause a shift in the secondary impedance, *Z*_*S*_, (Eq. S6) and thus a change in the reflected impedance, *Z*_*r*_ (Eq. 5). This then is detected wirelessly as a change in the total input impedance, *Z*_*IN*_, from the primary side (Eq. 6).

By inspecting Eqs. 2 and 3, reducing the distance between the coils (i.e., *x* → 0), should result in larger gains in voltage dropped over the DEP device due to a larger coupling coefficient, *k*. However, when operating on resonance, the coupling can become especially sensitive to position^25^ and result in a strong-coupling regime. This causes a frequency splitting of the WPT transfer function away from the resonant operating frequency, ω_0_, and thus overall performance is reduced^26^. Experimentally, this strong-coupling regime was demonstrated on a resonant circuit with our coplanar DEP device by sweeping the operating frequency at several distances of separation between the coils, see Fig. 3b–f. This splitting in the resonant frequency motivates the importance for avoiding the strong-coupling regime under this wireless trap and detect architecture. Equations 2–6 were used to empirically fit our experimental results using a nonlinear least squares approximation with the coupling coefficient, *k*, and particle load impedance, *Z*_*L*_, as fitting parameters, see Methods section and Supplementary Materials for more details.

### Demonstration of remote particle collection using WPT

**Fig. 4:**
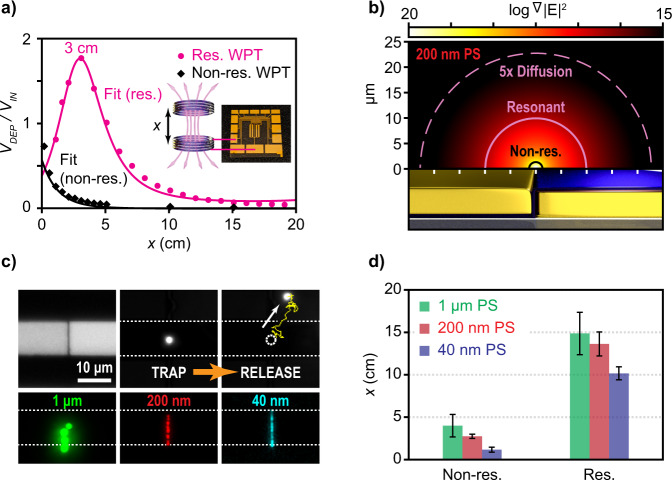
Long-range wireless DEP trapping experiment. **a** The optimal trapping distance, x, for resonant wireless power transfer (WPT)—using the coplanar DEP electrodes in solution, was measured at 3 cm. For non-resonant WPT, the voltage is maximized at x = 0 cm. Using a nonlinear least squares fit of the coupling coefficient, the resonant and non-resonant wireless voltage gain across the coplanar electrodes could simultaneously be fit, see Supplementary Materials. **b** A simulation comparing the trapping radius for 200 nm PS particles using non-resonant and resonant WPT at a distance of x = 3 cm (3.5 V_RMS_, 1 MHz input signal). The resonant trapping radius (solid pink, 9.9 µm) is 7.6× longer than the non-resonant (black, 1.3 µm) trapping radius equating to a trapping volume that will be 58× bigger. Likewise, the trapping radius in which DEP can collect 200 nm PS particles 5× faster than diffusion at resonance (pink dashed line, 22.7 µm) is 17.5× longer than the non-resonant radius which equates to a trapping volume that is 305× larger. The radial distribution of the gradient of the E-field squared is plotted for reference. **c** Microscope images of the coplanar DEP device during wireless trapping of 1 µm, 200 nm, and 40 nm PS particles. The top left image is a bright-field image of the DEP coplanar device. The other top images depict wireless trapping and releasing of a 1 µm particle as the coil separation is increased beyond sufficient power coupling for trapping. The bottom three fluorescent images show the trap site loaded with their indicated particle size. See Supplementary Materials and Figure S4 for trap and release data of the 200 nm and 40 nm PS beads. **d** Experimental results comparing the maximal coil separation, x, in which trapping of 1 µm, 200 nm, and 40 nm PS particles could be maintained for non-resonant and resonant operation. The distance between the coils is nearly 3× farther when using resonant operation. Error bars indicate ±one standard deviation.

Robust wireless particle collection was developed and characterized using a coplanar electrode device and in-house built primary and secondary inductive coils (detailed fabrication steps for both are outlined in the Method’s section). As mentioned prior, the WPT circuits were designed to resonant at 1 MHz for pDEP of PS particles. To compare the coupling efficiency, two WPT circuits were made in which one was resonant and the other non-resonant at 1 MHz. The voltage coupling over the DEP device was measured as a function of coil separation, *x*, between the primary and secondary inductor. For non-resonant operation, strong coupling was not observed and thus maximum coupling occurred when the distance between the coils was minimized (Fig. 4a). For resonant operation, the optimal coil separation was found experimentally at 3 cm where a gain of 1.8× the nominal input voltage was dropped over the device (Fig. 4a). Depending on the application, this gain can either offer larger trapping volumes for more particle collection (Fig. 4b) or robust trapping of particles over larger distance of separation (Fig. 4d) between the primary and secondary inductors as the coupling coefficient, *k*, reduces with distance, *x*. Characterized without a particle load, *Z*_*L*_, a nonlinear least squares fit of the coupling coefficient was found as a function of *x* (see Supplementary Materials) and resulted in the simultaneous fit for both the resonant and non-resonant voltage gain plots in Fig. 4a.

When considering the effects of resonant WPT on the trapping volume, this volume is classically defined as the volume in which the DEP force (Eq. 1) exceeds thermal Brownian motion (i.e. thermal trapping volume), see Supplementary Materials section. DEP, however, can interact with particles beyond this volume by applying a constant net drift to the particle’s random walk. Therefore, the trapping volume can be extended to include volumes where DEP transports a particle *n*× faster than diffusion over the equivalent distance, see Supplementary Materials section. The shape of these trapping volumes assuming semi-infinite coplanar electrodes is cylindrical due to the radial decay of the fringe E-fields from the nanogap and thus the radius of this cylinder can be used as a metric for defining the trapping volume size (Fig. 4b). Simulation of the relative trapping radii for PS particles ranging in size of 40 nm–1 µm were compared at the optimal coil separation of 3 cm for the resonant and non-resonant circuit using a 3.5 V_RMS_, 1 MHz signal. The thermal trapping radius and a 5× faster than diffusion (i.e., *n* = 5) trapping radius were calculated for each particle and tabulated in Table 1. An example surface plot of the different trapping radii for the 200 nm PS particles is depicted in Fig. 4b. Under these conditions, using resonant WPT theoretically yields thermal trapping volumes approximately ~58× greater (i.e. an average 7.6× longer trapping radii) than a non-resonant WPT trapping volume and 200–300× bigger when considering the radius in which collection is 5× faster than diffusion (Fig. 4b), see the Methods section for details.

**Table 1:** Comparison of the wireless trapping radii for non-resonant and resonant mode operation

Particle size (nm)	Non-resonant	Resonant	
	Thermal (µm)	Thermal (µm)	*n* = 5 (µm)
40	0.146	1.1	2.1
200	1.3	9.9	22.7
1000	11.3	86.5	>100

Simulations were made for several PS particle sizes in DI water using the experimental output voltage measured across the DEP device with an input signal of 3.5 V_RMS_ at 1 MHz and a 3 cm coil separation. It is observed that the resulting trapping volumes from these values is estimated to be 58× bigger when using resonant operation. The last column indicates the trapping volume in which DEP can collect particles 5× faster than diffusion (using resonant operation).

The implications on coil separation for stable trapping were tested experimentally by finding the furthest coil separation between the transmitting and receiving coil such that the target particle’s position remained fixed to the trap plane and velocity below that of thermal motion, see Methods and Supplementary Materials for more details. Keeping the source voltage to a digital logic value of 3.5 V_RMS_, the coils were brought close to one another to wirelessly load the trap with the target particles (Fig. 4c). Once the trap was loaded, the coils were then slowly separated from each other vertically until all the particles were able to thermally diffuse away from the trap site as observed using fluorescent imaging (Fig. 4c). The maximum distance of separation between coils was compared to a non-resonant case, in which the operating frequency for both was kept at 1 MHz (Fig. 4d) for pDEP on the PS beads. Resonant operation could hold stable trapping of particles with nearly 3× farther separation between the coils and could maintain trapping of particles as small as 40 nm up to 10 cm away using a digital voltage level RF signal (Fig. 4d).

### Concurrent wireless particle detection

**Fig. 5:**
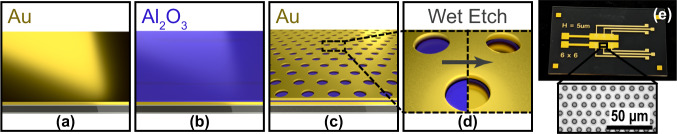
Metal-insulator-metal (MIM) nanogap device fabrication. Fabrication steps for the microhole array DEP device used for real-time wireless trapping and sensing experiments. **a** Step 1: Au is deposited to define the bottom electrode. **b** Step 2: A 20 nm Al_2_O_3_ layer is deposited using ALD to precisely define the electrode gap. **c** Step 3: A microhole pattern is defined using photolithography to increase the perimeter of active trapping sites. **d** Step 4: The exposed Al_2_O_3_ layer is removed using a wet etch process. This enables more fringe field to interact with the trapped particles. **e** A final device image of the microhole array DEP device. (Inset) A microsope image of the holes.

Next, simultaneous wireless detection of trapped particles was integrated for a dual-purpose platform. As the trapped particles replace the surrounding medium near the focused fringe fields of the nanogap—precisely positioned via DEP, the change in the dielectric permittivity and conductivity of this most sensitive volume will shift the device impedance. In order to increase the number of trapped particles for detection, the nanogap electrode device was modified to a stacked metal-insulator-metal (MIM) hole-array design with the same ALD-grown Al_2_O_3_ spacer film (20 nm thickness) defining the electrode nanogap (Fig. 5). With this hybrid design that combines metal-(ALD Al_2_O_3_)-metal stacked nanocapacitor with the periodic hole-array-based DEP electrode^27,28^, the number of nanogap junctions with exposed fringe fields were increased to offer more particle collection for sensing applications. The general fabrication process for this structure is also provided in the Supplementary Materials (Fig. 5). The size of the array was 600 × 600 µm and the total estimated trapping/edge length from the array and edges was 72.5 mm which is ~7000 longer than the previous coplanar structure. The increased trapping area resulted in a larger capacitance (C_DEP_ = 1.65 ± 0.02 nF, R_S_ = 15.5 ± 0.6 Ω, as measured in DI water at 1.3 MHz). A network analyzer was wired in a parallel tank circuit on the primary side to serve as both the wireless power supply and subsequent measurement instrument. The primary coil was kept the same but the corresponding parallel capacitor was chosen to optimize the new target operating frequency.

**Fig. 6:**
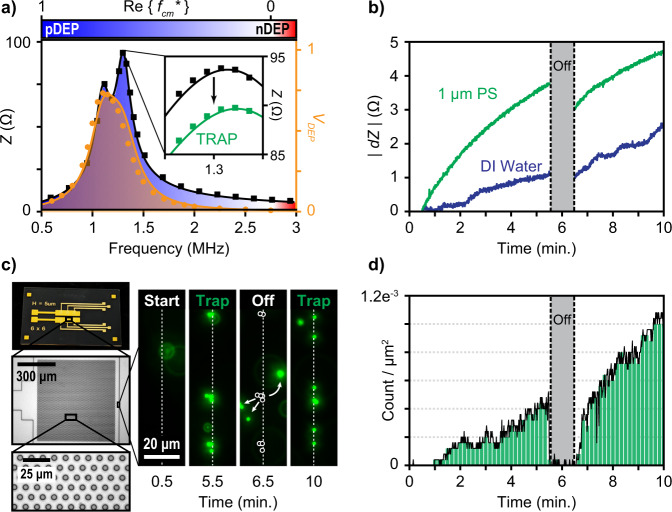
Wireless trapping with real-time sensing. **a** Before particle trapping, the impedance spectrum (black) was measured as a function of frequency with a coil separation of 2.5 cm. The regions where pDEP occurs is shaded blue (i.e. frequencies in which the real part of the CMF is greater than 0). The voltage amplitude coupled wirelessly over the microhole DEP device was recorded and peaked at 1.1 MHz (orange curve). These two curves were fit using nonlinear least squares with our measured circuit components. (Inset) The impedance spectrum over a focused region measured in which Z_IN_ was reduced from 94 to 89 Ω at the target frequency of 1.3 MHz after two minutes of particle trapping. **b** The change in absolute impedance was recorded on the transmitting circuit as a function of time for DI water and a solution containing 1 µm PS beads. A larger change in impedance is observed as more particles are collected. After 5 min, particles are released as the network analyzer is disconnected. Trapping resumes after 1 min. **c** Microhole array device and microscope images. Fluorescent images were taken at the array edge for clearly demonstrating trap and release events under the fluorescent microscope. Initially, no particles are observed along the indicated edge region of the array. After 5 min, particles have collected and were trapped on the array. They were then allowed to diffuse for 1 min before trapping once again. **d** The particle count taken from a 180 × 135 µm microscope field of view is plotted as a function of time. The particle count coincides with the observed change in impedance recorded in Fig. 6b.

The target operating frequency must meet three criteria. First, it should be within the range necessary for pDEP (as mentioned before) of the target particle as determined by its CMF (Eq. S1). For PS beads in DI water, this is any frequency within the blue shaded area of Fig. 6a (<2.58 MHz). Next, the frequency should couple sufficient voltage over the DEP device for DEP trapping as characterized by the circuit’s voltage transfer function, orange curve in Fig. 6a. Due to the change in device capacitance, the secondary coil was increased to 11 µH to shift the peak transfer function within the pDEP domain for the microhole device. The coil separation was fixed to 2.5 cm from the primary coil and the voltage drop across the DEP device was measured as a function of the wireless input frequency coupled from the network analyzer. The peak transfer was found at 1.1 MHz, see Fig. 6a. Lastly, the operating frequency should remain in a region where the total circuit’s input impedance spectrum, *Z*_*IN*_, (Eq. 6) is most sensitive to changes in the load impedance, *Z*_*L*_, of trapped particles (Eqs. 5 and S6). Using an LtSpice simulation, it was found that a primary parallel capacitor of 10.8 nF offered a sensitive change in impedance at a frequency of 1.3 MHz. At this frequency, the estimated CMF was 0.47 and the voltage dropped over the device was measured to be 0.53 V amplitude. Therefore, trapping at the 1.3 MHz frequency was sufficient for trapping 1 µm PS beads.

Using the prescribed device and circuit, the impedance spectrum was measured with the network analyzer (primary side) in DI water, see black curve in Fig. 6a. A nonlinear least squares fit that satisfied both the impedance spectrum (Eq. 6) and voltage transfer function (Eq. 3) using the circuit components provided above (i.e., *L*_*P*_ = 1.6 µH, *C*_*P*_ = 10.8 nF, *L*_*S*_ = 11 µH, *C*_*DEP*_ = 1.65 nF, *R*_*S*_ = 15.5 Ω, as measured at 1.3 MHz with the network analyzer measurement signal *V*_*IN*_ = 0.55 V, *R*_*int*_ = 50 Ω), see Fig. 6a. This was used to find the best fit values for the coupling coefficient and initial load impedance without particles, which resulted in *k* = 0.2 and *Z*_*L*_ = 1539.5 – 743.3*j* Ω, respectively. Then, a solution containing 1 µm PS fluorescent beads were placed onto the sample in which the power from the network analyzer’s measurement signal was sufficient to wirelessly trap the particles onto the microhole array (Fig. 6c). Subsequently, a change in the absolute value of *Z*_*IN*_ was observed by the network analyzer on the primary side (inset of Fig. 6a) in which this spectrum was taken after two minutes of trapping. Repeated a total of three times, *Z*_*IN*_ was reduced from 93.66 ± 0.05 Ω to 89.28 ± 0.01 Ω (one standard deviation) at the target frequency of 1.3 MHz. Keeping the same fitted coupling coefficient (*k* = 0.2), the new best fit of *Z*_*L*_ due to trapped particles was *Z*_*L*_ = 1969.5–294.5*j* Ω, with a difference in impedance of 430 + 448.8*j* Ω, see inset of Fig. 6a.

Furthermore, the time dependent change in the load impedance could be wirelessly recorded during DEP trapping. Initially, the impedance of the wireless circuit was measured on the transmitting side by the network analyzer with DI water only to gain a background signal (Fig. 6b). Then, this was repeated with the 1 µm PS fluorescent bead solution. The absolute change in *Z*_*IN*_ was recorded as a function of time with the network analyzer while visual confirmation of trapped particles was simultaneously recorded using fluorescent microscopy (Fig. 6b, c). After five minutes, the network analyzer was disconnected from the coil for one minute to allow the PS beads to be released and empty the microhole traps (Fig. 6c)—observed as a break in the measured *Z*_*IN*_ (Fig. 6b). Then the network analyzer was reconnected and wireless trapping and detection was again observed (Fig. 6b, c). The trapped particle count was recorded in time (Fig. 6d) within a microscope field-of-view of 180 × 135 µm (which represents 7% of the total sensing area). The particle count followed similar trends as the observed change in impedance (Fig. 6b and d), suggesting a correlation between the measured shift in impedance and number of particles present within the sensing volume.

## Discussion

By combining an efficient nanogap electrode array with resonant WPT, a single measurement signal is used to simultaneously collect and position suspended particles within the high-gradient fringe field regions of a nanogap capacitor and relay back the change in impedance wirelessly. This was demonstrated using inductive coupling between a parallel-series circuit architecture in which robust trap and release of virus-sized, nanoparticles could be maintained with a >8 cm separation between the coils using a low-voltage 3.5 V_RMS_ RF signal. Wireless detection of the impedance change from the rapidly trapped particles could be measured in real-time when using a network analyzer on the primary side to supply the trapping voltage. Our wireless trapping and sensing method would integrate especially well with point-of-care and aqueous applications where electrical power supply and detectors is not favorable—e.g., liquid tubing, microwell plates, wearable biotech, or possibly implantable technology.

We appreciate that each of those applications have unique acceptance criteria that would require more targeted designs to accommodate. For instance, the solution conductivity and capability to dilute would alter the allowable operating frequencies for pDEP and equivalent circuit design. If a pDEP regime cannot be found, alternative DEP architectures can be considered, including chemical tagging to metallic nanoparticles^29^, a nDEP quadrupole for particle collection^30–32^ or incorporating electrodynamic flow to focus the concentration of target analytes^33^. Already, wireless, electrofluidic transport of viral capsids and proteins in physiological buffer has been demonstrated where the sample solution, rather than the suspended particles are trapped^34^. One could fathom using a combined approach with the work presented herein to optimize the WPT for both delivering physiological buffer and wirelessly detecting suspended analytes where dilution or tagging is not desired. This work, however, overcomes the critical challenge of low-voltage particle trapping and concurrent wireless impedance detection with the goal of inspiring more advanced, point-of-care^35,36^, handheld^37^, and/or smartphone-based^38^ biosensing platforms.

## Methods

### Preparation of coil inductors

The coils for long-distance dielectrophoretic trapping were designed to compare resonant and non-resonant operations at 1 MHz operating frequency. The coils’ outer diameter was 3.5 cm, and inner diameter was 3 cm, five turns wound as the spiral type with 18 AWG magnetic copper wire. The primary coil was measured as *L*_*p*_ = 1.68 µH at 1 MHz and the 15 nF parallel compensated network capacitor was added for resonance at 1 MHz. The secondary coil was measured as *L*_*s*_ = 1.8 µH at 1 MHz and the 14 nF compensated network capacitor was added for resonance at 1 MHz.

Another coil was designed for wireless sensing using a parallel-series compensation network. The secondary side inductor was 20 turns wound to increase the inductance for sensing. The inner diameter was 0.5 cm, and the outer diameter was 5 cm as the spiral type with 20 AWG magnetic copper wire. The secondary coil was measured as *L*_*s*_ = 11 µH inductance. Based on the parallel-series compensation network design of the WPT circuit, the primary capacitor was designed considering the equivalent impedance of the DEP sensor. The compensation network was calculated using the following equation^39^.

7 $${C}_{p}=\frac{{L}_{P}}{{\left(\frac{{{w}_{0}}^{2}{M}^{2}}{{R}_{s}}\right)}^{2}+{{w}_{0}}^{2}{{L}_{p}}^{2}}$$

where *w*_*o*_ is the angular resonant frequency, *M* is the mutual inductance, *L*_*P*_ is the inductance of the primary coil, *R*_*s*_ is the equivalent resistance of the secondary side. The primary capacitor (*C*_*P*_) was designed as 10.8 nF under the *k* = 0.2, *R*_*S*_ = 15.5 Ω, and an inductance of the primary coil of *L*_*P*_ = 1.6 µH as measured at 1.3 MHz.

### Long-distance dielectrophoretic trapping and release

A sinusoidal AC signal (1 MHz, 3.5 V_RMS_) was applied across the transmitting LCR parallel circuit (*C*_*P*_ = 15 nF, *L*_*P*_ = 1.68 µH) and was coupled to the secondary LCR circuit (*L*_*S*_ = 1.8 µH) in which a parallel capacitor was wired to the coplanar DEP electrode device. For the resonant WPT circuit, the total *C*_*DEP*_ = 14.022 nF (22 pF DEP device in parallel with an additional 14 nF) and the non-resonant WPT total *C*_*DEP*_ = 37 pF (22 pF DEP device in parallel with an additional 15 pF). The coil inductors were coaxially aligned using a rod and clamps and the distance could be varied by sliding the transmitting coil inductor along the length of the rod. Fluorescent imaging of the nanogap was recorded using a 50× microscope objective (NA 0.55, Nikon) to confirm trapping of particles. Three different sized polystyrene (PS) particles that were fluorescently labeled (Bangs Labs) were tested for wireless trapping. The particle sizes tested were: 1 µm (1.06 mg/mL or 28.2 fM), 200 nm (0.1 mg/mL or 353 fM), and 40 nm (0.1 mg/mL or 40.8 pM). Each was mixed into its own DI solution with a measured conductivity of 4 × 10^−4^ S/m (measured by B-771 LAQUAtwin, Horiba Scientific). For both resonant and non-resonant operation, the transmitting inductive coil was brought near the secondary inductive coil until the 10 µm long trapping coplanar gap was filled with particles (Fig. 4c). Then the distance between the coils was increased 1 cm for resonant operation and 0.25 cm for non-resonant operation every ~60 s, respectively, until all the particles were released. The measured voltage across the DEP device and coil separation were than recorded. Resonant and non-resonant wireless trapping and release was repeated across three different devices for each of the three particle sizes (Fig. 4d). Demonstration of releasing a 1 µm PS particle is shown in Fig. 4c and demonstration of the release of the 200 nm and 40 nm PS particles is included in the Supplementary Materials and Fig. S4. Using a nonlinear least square fit of the coupling coefficient, *k* (Eq. 2), the experimental voltage gain for resonant and non-resonant WPT could be fit (Fig. 4a), see Supplementary Materials for fitting of the coupling coefficient.

### Wireless sensing

Per the target conditions described in the main text operating at 1.3 MHz, the transmitting LCR (*C*_*P*_ = 10.8 nF, *L*_*P*_ = 1.6 µH) was wired to a network analyzer, which simultaneously power wireless trapping and measured the impedance change on the transmitting side. The secondary coil (*L*_*S*_ = 11 µH, *R*_*S*_ = 15.5 Ω) was wired to the microhole array DEP device (*C*_*DEP*_ = 1.65 nF) and fixed 2.5 cm from the transmitting inductor coaxially. The impedance spectrum, *Z*_*IN*_, and voltage drop, *V*_*DEP*_, over the microhole array was recorded as a function of frequency (500 kHz–3 MHz) in DI in which the voltage was recorded using an oscilloscope to observe the voltage transfer function (orange line in Fig. 6a). A focused measurement spanning 1.2–1.4 MHz of the impedance spectrum was recorded three times in DI water and three times after two minutes of particle trapping (inset of Fig. 6a).

Time dynamics was recorded using the network analyzer fixed at 1.3 MHz. Initially, DI water was placed on the DEP device (Fig. 6b). The input impedance and phase on the transmitting circuit was recorded in time (sampled every 0.5 s) for 10 min. After 5 min, the network analyzer was disconnected from the transmitting coil to de-power the device for 1 min before reconnecting. This was done to record a baseline signal for the PS trapping experiment. Next the device was dried under a stream of N_2_ and a solution containing 1 µm (1.06 mg/mL or 28.2 fM) was introduced to the device. Fluorescent imaging with 1 s frames (2 × 2 pixel binning, 400 ms exposure; Micro-Manager) using a charge-coupled device (CCD) camera (CoolSNAP HQ2, Photometrics) and the same 50× microscope objective (NA 0.55, Nikon) was used to record particle position and trapping events. A 30 s baseline was recorded before trapping to characterize the particle diffusion velocity before trapping. Using the ImageJ Trackmate Software, the average absolute particle diffusion velocity before trapping was 1.084 ± 0.816 µm/s. Then, the network analyzer was applied to the primary circuit and the input impedance and phase was again recorded in time for 10 min (sampled every 0.5 s) with trapping events simultaneously recorded over the microscope field-of-view (180 × 135 µm) (Fig. 6c). After 5 min, the network analyzer was disconnected and the trapped particles were released and began to diffuse away from the sensing surface as observed under fluorescent microscopy (Fig. 6c). After 1 min, the network analyzer was reconnected back to the primary circuit and particle trapping restarted. The number of particles with an absolute velocity <73 nm/s (i.e., three standard deviations below the diffusion velocity) were considered trapped and counted as a function of time during the impedance sensing experiment (Fig. 6d). It was observed that 88.7% of the counted particles during these 5 min met this criterion within the field-of-view, resulting in the histogram of the absolute particle velocity to shift towards zero, see Supplementary Materials. While this field-of-view represents only 7% of the total sensing area, the trapped particle count follows the same trends as the impedance shift measured during trapping and release (Fig. 6b and d).

## Supplementary information

Supplementary Information

## Data Availability

Most data generated or analyzed during this study are included in the published article or Supplementary Material. Raw data lists will be made available by the corresponding author on request.
