# Supplementary material for: Wireless dielectrophoresis trapping and remote impedance sensing via resonant wireless power transfer
Source: Nat Commun. 2023 Jan 6;14:103. doi: 10.1038/s41467-022-35777-2 (PMC9821345; doi:10.1038/s41467-022-35777-2)
Supplement: Supplementary file 1 — Supplementary Information [file 41467_2022_35777_MOESM1_ESM.pdf]

Supporting Information

Wireless dielectrophoresis trapping and remote impedance sensing via resonant wireless power transfer

Christopher T. Ertsgaard, Minki Kim, Jungwon Choi and Sang-Hyun Oh*

Department of Electrical and Computer Engineering, University of Minnesota,

Minneapolis, Minnesota 55455, United States

*E-mail: sang@umn.edu

Clausius-Mossotti factor

As mentioned in the main text, two regimes of DEP are defined: positive DEP (pDEP) in which particles migrate towards increasing E-field gradient (and thus the sensitive fringe fields) and negative DEP (nDEP) in which particles are repelled from the fringe E-fields. The factor determining whether a particle experiences pDEP or nDEP for a given frequency is contained within the Clausius-Mossotti factor, $f_{CM}*$, (CMF) in which the angular frequency (ω) of the AC signal and the complex dielectric permittivity of the particle and the surrounding solution (ε_m* and ε_p*, respectively) determine its value (Equation S1).

$$f_{CM}^{*}(\omega) = \frac{\varepsilon_p^{*}(\omega) - \varepsilon_m^{*}(\omega)}{\varepsilon_p^{*}(\omega) + 2\varepsilon_m^{*}(\omega)} \quad \text{(S1)}$$

The complex permittivity can be approximated using the following low-frequency approximation:[1,2]

$$\varepsilon^{*}(\omega) = \varepsilon - \frac{i\sigma}{\omega} \quad \text{(S2)}$$

where, i, is the imaginary unit, and ε and σ is the material's dielectric constant and electrical conductivity, respectively. The sign on the real part of the CMF (Equation S1) determines whether the operation is within the positive or negative DEP regimes. The CMF of Polystyrene beads in DI water, which were used as model particles in all experiments, is plotted in Figure S1 and the cross-over frequency from pDEP to nDEP is predicted to be 2.58 MHz. For sensing, pDEP will be the target operating regime such that particles are trapped close to the sensing electrodes and thus an operating frequency below this crossover frequency should be used.

Defining the trapping radius/volume

The maximum radial distance from the electrodes in which DEP can collect particles is referred to as the trapping radius. It is a defining parameter of the trapping volume. Classically, this trapping radius is defined as the distance in which the DEP force (Equation 1) is greater than the 1D thermal force of Brownian motion,[2-4] see Equation S3

$$F_{DEP} > \frac{kT}{2R} \tag{S3}$$

Here, T, is the temperature of the ambient solution, k, is Boltzmann's constant, and R, is the radius of the spherical particle.[2-4] The distance from the gap at which the gradient of the E-field squared satisfies this condition can be solved for (Equation S4) and defines the thermal trapping radius.

$$\nabla|\boldsymbol{E}|^2 > \frac{k\,T}{4\,\pi\,\varepsilon_m\,R^4\,Re\{f_{CM}^*\}} \tag{S4}$$

Here, ε_m, is the dielectric constant of the ambient solution and, $f_{cm}{}^*$ is the CMF defined in Equation S1. However, DEP can still exhibit a net bias on particles beyond this distance such that the net movement of the particles towards the gap is n-times faster than diffusion would naturally deliver the particles. This condition on the gradient of the E-field squared becomes the following:[2]

$$\nabla|\boldsymbol{E}|^2 > \frac{n\,k\,T}{T_R\,\pi\,\varepsilon_m\,R^3\,Re\{f_{CM}^*\}} \tag{S5}$$

Where, T_R, is the distance from the gap in which the gradient of the E-field is evaluated. For larger values of n, the resulting trapping radius at which the E-field satisfies the condition of Equation S5 will be reduced.

Circuit Derivation

The secondary circuit impedance, Z_S, formed by the DEP device capacitance, C_{DEP}, and the secondary inductor, L_S, forms a series LCR with a series resistance, R_S, inductive reactance, X_{LS}, and DEP device capacitive reactance, X_{DEP}. The capacitive reactance is then in parallel with the load impedance, Z_L, which changes in time as particles are trapped, see Equation S6.

$$Z_S = R_\mathrm{S} + jX_\mathrm{LS} + \frac{jX_{DEP}\, Z_L}{jX_{DEP} + Z_L} \tag{S6}$$

Therefore, the current in the primary, parallel LC configuration (Figure 3a) coupled to a secondary series LCR becomes the following:

$$I_P = \frac{V_{IN}}{Z_{IN}}\left(\frac{jX_{CP}}{jX_{CP} + jX_{LP} + Z_r}\right) \tag{S7}$$

where X_{LP} and X_{CP} are the reactance of the primary inductor and capacitor, respectively. The input voltage, V_{IN}, from the power source then sees a total input impedance, Z_{IN}, (Equation 6) which includes its internal resistance, R_int, (typically standardized to 50 Ω).

Fitting of the coupling coefficient

In order to fit the voltage transfer function data as a function of coil separation, x, (Figure 4a), the coupling coefficient, k, must first be fit as a function of x. The coupling coefficient was approximated as an exponential decay function with coil separation using the fitting parameters, a, b, c and d, see Equation S8.

$$k = a\, e^{bx^2+cx} + d \tag{S8}$$

Equation 2 of the main text relates this coupling coefficient to the mutual inductance, M, and thus can be used to fit the experimentally found voltage gain, V_{DEP}/V_{IN}, of Figure 4a. No particle load, Z_L, was used when characterizing the transfer function and thus the secondary circuit impedance was: $Z_S = R_S + X_{LS} + X_{DEP}$ (R_S is the series resistance, X_{LS} is the inductive reactance, and X_{DEP} is the DEP device capacitive reactance). An operating frequency of 1 MHz was used during data collection for Figure 4a (V_{IN} = 0.707 V_{RMS}) with the following circuit parameters: R_{int} = 50 Ω, C_P = 15 nF, L_P = 1.68 µH, L_S = 1.8 µH, and R_S = 0.2 Ω (see circuit diagram in Figure 3a). The resonant wireless power transfer (WPT) circuit had a C_{DEP} = 14.022 nF (22 pF DEP device in parallel with an additional 14 nF) and the non-resonant WPT had a C_{DEP} = 37 pF (22 pF DEP device in parallel with an additional 15 pF). Using a Non-linear least squares fitting function in MATALB, Equation S8 was fit using Equations 3-6 and the circuit parameters provided above to the experimental data found in Figure 4a. This resulted in the following fitting parameters: a = 0.6, b = 244 m^{-2}, c = -81.5 m, and d = 0.0009. The exponential function of the coupling coeffect, k, as a function of distance using these parameters is provided, see Figure S2. This function of k then provided the simultaneous fit for both the resonant and non-resonant data in Figure 4a.

Absolute particle velocity distribution with and without wireless trapping

Prior to wireless trapping and detection, a 30 s baseline recording of the particle velocity was made using fluorescent imaging with 1 s frames (2 × 2 pixel binning, 400 ms exposure; Micro-Manager). Suspended 1 µm polystyrene beads (Bangs Labs) were seen to randomly diffuse prior to wireless actuation with an average absolute particle diffusion velocity of 1.084 ± 0.816 µm/s,

see Figure S3. Then, after applying the Network analyzer to the transmitting coil, particles were wirelessly trapped resulting in the majority of the particle velocities being reduced to ~15 nm/s, see Figure S3. Particles were considered trapped if they held a velocity less than three standard deviations below the diffusion velocity. Over five minutes of trapping, 88.7% of the recorded particles held a velocity below 73 nm/s and were considered trapped. The number below this threshold were counted at each timepoint and used to plot Figure 4d.

Device Fabrication Steps

The fabrication steps for the coplanar nanogap devices were developed and optimized in our previous work and follow these general steps (Figure 2). First, 500 µm thick Borofloat 33 glass wafers (University Wafer) are cleaned in a standard piranha solution and rinsed thoroughly with deionized (DI) water. A photolithography step defines the first edge of the nanogap electrode which is deposited using Au electron-evaporation deposition (150 nm Au, with a 3 nm Chromium adhesion layer) (STEP 1, Figure 2a). After a lift-off process, an atomic-layer deposition (ALD) of alumina (Al_2O_3) is deposited over the first electrode and will define the nanogaps width. A nominal value of 20 nm for all experiments was used (STEP 2, Figure 2b). After ALD, a non-conformal Au evaporation is deposited (with no adhesion layer) to create the second electrode (130 nm Au) (STEP 3, Figure 2c). The excess Au layer not connected to the second electrode edge is removed using adhesive tape (STEP 4, Figure 2d). Multiple, 10 µm coplanar electrode devices were then defined along the length of the nanogap using negative resist photolithography. Finally, ion milling was used to etch out and isolate each device. The device capacitance was characterized over five devices each measured independently three times. In an air environment the coplanar devices had an average capacitance and standard deviation of 19.7 ± 0.6 pF. In a DI water environment, the

capacitance increased to 22.2 ± 0.8 pF. These values were measured using the Network Analyzer at the operating frequency of 1 MHz.

The stacked microhole array nanogap device was fabricated using this general protocol (Figure 5). First, 500 µm thick Borofloat 33 glass wafers (University Wafer) are cleaned in a standard piranha solution and rinsed thoroughly with DI water. A 100 nm layer of Au (with 5 nm Ti adhesion layer) is sputtered unto the wafer and a photolithography step defines the shapes of the bottom electrode (STEP 1, Figure 5a). Ion milling was then used to etch out and isolate each device which was then thoroughly cleaned in 1165 Microposit Remover, acetone, methanol, and isopropyl alcohol. Next, a 20 nm Al_2O_3 was deposited over all the devices and defined the nanogap thickness (STEP 2, Figure 5b). A microhole array was patterned using photolithography. The microholes were 5 µm in diameter with a 10 µm pitch and the entire array covered 600 × 600 µm square area (STEP 3, Figure 5c). A 200 nm layer of Au (with 5 nm Ti adhesion) was then deposited using electron evaporation to define the top electrode. After lift-off, the exposed Al_2O_3 layer within the microholes were etched using phosphoric acid at 50 C for 4 minutes to increase the device sensitivity. Finally, in order to make a low resistance electrical connection with the inductive coils, SMA connectors were soldered to the device. The device capacitance and series resistance was characterized over two devices each measured independently three times. In an air environment the average capacitance and standard deviation measured was 1.418 ± 0.001 nF and the average series resistance was 10.0 ± 0.2 Ω. In a DI water environment, the capacitance increased to 1.645 ± 0.022 nF and the series resistance to 15.5 ± 0.6 Ω. These values were measured using the Network Analyzer at the sensing operating frequency of 1.3 MHz.

Trap and release demonstration of 200 nm and 40 nm PS beads

In addition to the trap and release of 1 μm particles described in the main text, the trap and release function was also demonstrated for the smaller 200 nm and 40 nm PS beads, however, wireless detection was only demonstrated for the 1 μm particles. As mentioned in the Method's section, the long-distance trapping experiment of Figure 4d involves loading a trap and then increasing the distance between the coils until all particles were released. Example of the trapping and release of the sub-micron particles shown in Figures 4c are presented in Figure S4. In both cases, the coplanar DEP device was used, and images were false colored red for 200 nm and cyan for 40 nm PS beads to differentiate the different sized particles under test. Initially, the traps are recorded as empty for a baseline measurement. Then a resonant wireless 3.5 V_{RMS} (1 MHz) signal is applied over a distance of 10 cm or less and 7 cm or less for the 200 nm and 40 nm particles, respectively. After the traps are loaded for approximately three minutes, the distance between the coils is increased to 13 cm and 8.5 cm for the 200 nm and 40 nm particles, respectively. At these distances, the traps became weaker than the thermal Brownian motion for the trapped particles and thus are released, see Figure S4. After increasing the coils to 14 cm and 10 cm for the 200 nm and 40 nm PS beads, respectively, the traps are completely emptied. In this way, one can wirelessly manipulate the trapping and release of suspended particles as small as 40 nm.

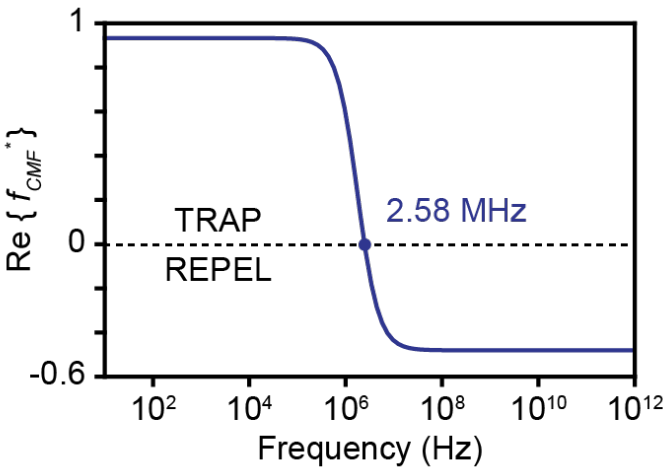

Figure S1. **Clausius-Mossotti Factor (CMF) for polystyrene beads in deionized water.** The real part of the CMF for polystyrene (PS) beads in a deionized (DI) water environment was simulated using Equations S1-S2. The DI water conductivity was measured in lab to be $\sigma = 4 \times 10^{-4}$ S/m and a dielectric constant of $\varepsilon_m = 80\ \varepsilon_0$ was approximated. The PS beads were approximated to be $\sigma = 160 \times 10^{-4}$ S/m with a dielectric constant of $\varepsilon_p = 2.56\ \varepsilon_0$.[2] These values were used to define the complex dielectric partitivities $\varepsilon_m^*(\omega)$ and $\varepsilon_p^*(\omega)$ (Equation S2), respectively. According to this simulation, frequencies greater than ~2.58 MHz will result in negative DEP and thus the PS beads will be repelled from the sensitive fringe fields. Therefore, operating frequencies less than this will result in positive DEP trapping and be the upper bound on the operating frequency for circuit design.

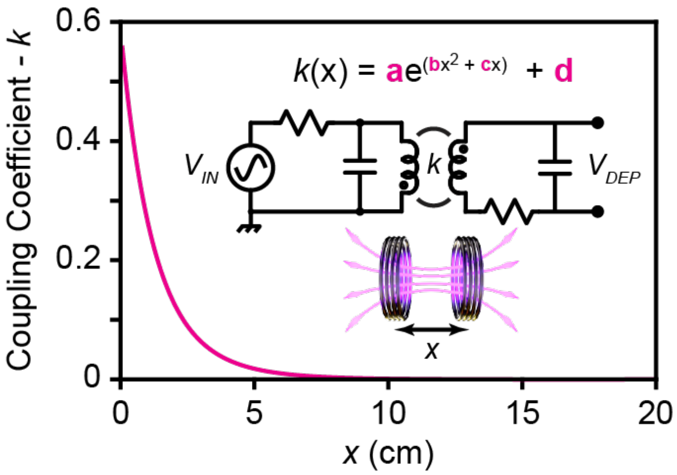

Figure S2. **Non-linear least squares fit of the coupling coefficient for resonant and non-resonant wireless power transfer (WPT).** Using the measured voltage gain in Figure 4a, the coupling coefficient, k, was fit as a function of coil separation, x (Equation S8). Using a non-linear least squares fit for k, the following parameters were found: $a = 0.6$, $b = 244$ m^{-2}, $c = -81.5$ m, and $d = 0.0009$. This resulted in the following exponential function for k and the simultaneous fit of the experimental voltage gain for both the resonant wireless power transfer (WPT) and non-resonant WPT circuits used in Figure 4a.

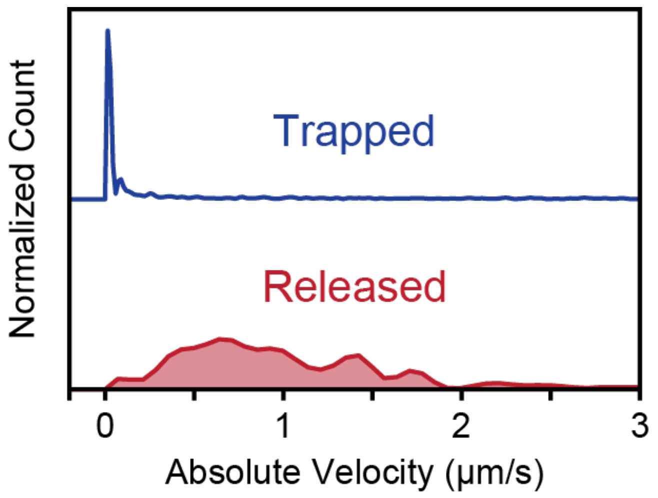

Figure S3. **Absolute particle velocity histogram.** The red curve is the absolute velocity of the suspended 1 μm particles averaged over 30 s before DEP trapping was initiated. During this period, particles are released and their velocity is diffusion limited. The average velocity was 1.084 ± 0.816 μm/s. After applying the Network analyzer for wireless DEP trapping, the absolute velocity was averaged over five minutes of trapping (blue curve). The new histogram shifts towards zero as the particle velocities approach zero when trapped. The average was 0.710 ± 1.300 μm/s with the majority of the population at 15 nm/s. It was found 88.7% of the particles had a velocity less than 73 nm/s. This is three standard deviations less than the diffusion mean velocity and thus was defined as trapped for particle counting.

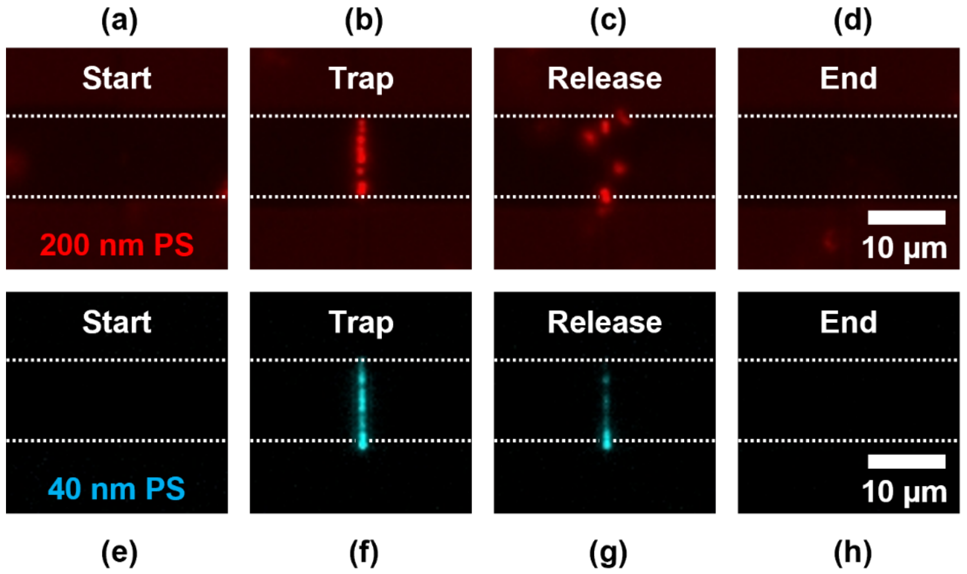

Figure S4. **Wireless Release of smaller particles.** Trap and release experiments were demonstrated using a coplanar DEP device for 200 nm (red) and 40 nm (cyan) PS beads in DI water. Images were false colored to differentiate the different sized particles tested. **(a)** Initially, the trap is empty of particles. **(b)** After wirelessly applying a resonant 3.5 V_{RMS} (1 MHz) signal over a distance less than 10 cm for three minutes, the nanogap capacitor is well loaded with trapped 200 nm PS particles. **(c)** When the separation between the coils is increased to 13 cm, the trap is overcome by thermal diffusion and particles are released from the trap. **(d)** Trap is fully emptied when the distance between coils reaches 14 cm. **(e)** Trap and release experiment is repeated for 40 nm PS beads in DI water using a different coplanar DEP device. Due to the smaller particles exhibiting lower fluorescence, a background image was subtracted from all frames to differentiate from background noise. Initially, the trap is empty. **(f)** After wirelessly applying a resonant 3.5 V_{RMS} (1 MHz) signal over a distance less than 7 cm for 3.5 minutes, the nanogap capacitor is well loaded with trapped 40 nm PS particles. Since the thermal forces are stronger for the smaller particles, the coils must be closer to couple more voltage across the nanogap capacitor to load the trap. **(g)** When the separation between the coils is increased to 8.5 cm, the trap is overcome by thermal diffusion and particles begin to be released from the trap. **(h)** Trap is fully emptied when the distance between coils reaches 10 cm.

Supplementary Material References

1 Mehrotra, P., Chatterjee, B. & Sen, S. EM-Wave Biosensors: A Review of RF, Microwave, mm-Wave and Optical Sensing. *Sensors* **19**, 1013, doi:10.3390/s19051013 (2019).

2 Morgan, H. & Green, N. G. *AC electrokinetics*. 1st edn, (Research Studies Press Ltd, 2003).

3 Holzel, R., Calander, N., Chiragwandi, Z., Willander, M. & Bier, F. F. Trapping single molecules by dielectrophoresis. *Physical Review Letters* **95**, 128102, doi:10.1103/PhysRevLett.95.128102 (2005).

4 Barik, A., Chen, X. S. & Oh, S. H. Ultralow-Power Electronic Trapping of Nanoparticles with Sub-10 nm Gold Nanogap Electrodes. *Nano Letters* **16**, 6317-6324, doi:10.1021/acs.nanolett.6b02690 (2016).
